# Physical Activity Levels in Chinese One-Year-Old Children and Their Parents, an Early STOPP China Study

**DOI:** 10.1371/journal.pone.0153605

**Published:** 2016-04-14

**Authors:** Hong Mei, Elin Johansson, Maria Hagströmer, Yuelin Xiong, Lanlan Zhang, Jianduan Zhang, Claude Marcus

**Affiliations:** 1 Department of Maternal and Child Health Care, School of Public Health, Tongji Medical College, Huazhong University of Science and Technology, Wuhan, Hubei, China; 2 Division of Pediatrics, Karolinska Institutet, Department of Clinical Science, Technology and Intervention, Stockholm, Sweden; 3 Division of Physiotherapy, Karolinska Institutet, Department of Neurobiology, Care Sciences and Society, Stockholm, Sweden; Karolinska Institutet, SWEDEN

## Abstract

**Background:**

Physical activity (PA) is associated with health benefits, already in childhood. However, little is known about actual levels, patterns and gender differences in PA level in very young children. This study examines Chinese one-year-old children and their parents’ PA levels and patterns, and assesses the correlations between children’s PA level and gender, body mass index standard deviation score (BMI SDS), parental BMI and parental PA level.

**Methods:**

Data from 123 families participating in the Early STOPP China study were used. Families were recruited based on parental BMI and were classified as either high-risk or low-risk of obesity. Parents and children wore an ActiGraph GT3X+ to assess the average PA levels. PA levels and hourly patterns during weekdays and weekends were examined as were correlations with gender, BMI SDS, parental BMI and parental PA levels.

**Results:**

There were no significant differences in children’s averaged PA between risk groups, genders, or between weekdays and weekends. Children’s peak average activity level was at 7 pm and they were least active at 3 pm (*p*<0.001). Both mothers and fathers demonstrated a similar PA pattern as their children, although paternal PA level was consistently lower than that of mothers and children. No significant association was found between children’s PA and their gender, BMI SDS, parental BMI or paternal PA levels. Maternal PA was found positively associated with child PA (p<0.05).

**Conclusion:**

PA in one-year-old Chinese children vary over the day but weekdays and weekends are similar. At this age, children’s PA is not related to gender, BMI SDS, parental BMI or paternal PA. Larger scale studies with more contextual information are needed to improve the understanding of our findings.

## Introduction

Childhood obesity is a major public health concern worldwide and has been increasing rapidly in China. The prevalence of obesity among infants and preschool children in China increased from 0.5% in 1996 to 1% in 2006 [1] and became 10.1% for preschool children in 2014 [2]. As obesity in childhood increases the risk for obesity in adulthood [3], later cardiovascular disease [4] and psychosocial and behavioral complications [5], preventing obesity in children is a worldwide health priority. Childhood obesity is multi-factorial and parental obesity has been shown to be the most important risk factor [6, 7]. Children growing up with one overweight parent have a double risk of becoming obese and if both parents are obese the risk is further increased [8], probability due to the shared genetic proposition and obesogenic environmental factors. Therefore, promoting a healthy lifestyle in coping with the obesogenic environment is of specific importance for children at high risk of obesity [9]. Results from studies on preschool [10] and primary school children [11] show that physical activity (PA) can contribute to the reduction of childhood obesity [12]. Thus the health implications of PA during the early years cannot be overlooked. Parents play a crucial role in forming PA habits in their preschool children and a parental-child association in PA has been shown [13]. Yet, it is not known if this association is dependent on parental weight status.

Studies of PA in children under the age of three are sparse [14, 15], probably due to the difficulties related with PA assessment. Young children per se, are not able to recall past activities, and it is difficult for adults to assess their activity due to the intermittent nature of children’s PA [16]. Thus, objective methods, such as accelerometry, are preferable. Accelerometers have traditionally been worn on the waist but a wrist placement has been found to increase compliance substantially [17, 18]. Wrist-mounted accelerometers have been found valid and feasible for use in adults [19] as well as in very young children [20].

Early STOPP China is a cohort study, in which children are annually followed up from age 1 to 6 years, together with their parents [21]. This enables the identification of behaviors that promote health or contribute to prevention of obesity, such as PA, diet and sleep in children with normal weight and overweight or obese parents. The primary objective of this sub-study was to examine accelerometry-measured PA levels and patterns in one-year-old Chinese children and their parents. A further aim was to assess the associations between children’s PA with gender, body mass index (BMI), parental weight status and parental PA levels.

## Methods

### Participants

Baseline data from participants in the Early STOPP China was used. Families (n = 299) living in the Hankou and Hanyang districts of Wuhan city were recruited through the Child Systematic Health Service Center (CSHSC) before the child’s first birthday. Inclusion criteria were: 1) the parents were registered residents of Wuhan city; 2) at least one child in the household was younger than one year old at the time of recruitment; 3) the child had no chronic health condition that could potentially affect growth, PA or dietary patterns. Candidate high-risk (HR) and low-risk (LR) families were recruited based on parental BMI. As Chinese population have a higher percentage of body fat compared to Western people at the same BMI, lower cut-offs were used for the Chinese parents [22]. Families with both overweight (BMI = 24 kg/m^2^—<28 kg/m^2^) parents or at least one obese parent (BMI ≥28 kg/m^2^) were included in the HR group, while families of two normal-weight parents (BMI <24 kg/m^2^) or one normal-weight and one overweight parent were allocated to the LR group.

The study was approved by the Ethical Committee (IORG0003571) of Tongji Medical College, Huazhong University of Science and Technology in Wuhan, China.

### Procedures

The parental pre-pregnancy weight and height were collected by trained staff in the CSHSC for those children born from December 2010 to November 2012. Thereafter the parental BMI was calculated and candidate HR and LR families were identified according to parental BMI status. After that, families were contacted by telephone to explain the objectives and procedure of the project. For those families who consent to participate, home visits by two trained research assistants were conducted within 15 days of the child’s first birthday. Mother, father and child were each fitted with an accelerometer (ActiGraph GT3X+) on their left wrist. The parents were asked to wear the monitors for seven consecutive days and nights without removal, except for during bathing. Oral and written instructions regarding the usage of the monitors were provided to the families. A written inform consent was collected on site.

Parents filled in questionnaires regarding maternal weight gain during pregnancy, type of child care (mother/father/grandparents) and child gross motor development (i.e. whether the child could walk or not). Socio-economic status was determined by educational level (middle school, high school or college/university), household income (≤3000, 3001–5000, 5001–10000, ≥10001 RMB/month) and living space (m^2^). Parents also answered a question about their employment status. Child weight and length at birth were obtained from the Children Health Care Booklet held by the parents. In order to separate sleep time at night from daily PA, the sleeping patterns of both parents (bed and wake-up time for the previous month) and child (seven-day’s night time sleep record) were recorded by the parents. Weight was measured in both parents and children to the nearest 0.1 kg using an electronic scale (Xiangshan EB9272H), with the subjects wearing light indoor clothing. Recumbent length of the child was measured to the nearest 0.1 cm using a portable infant length rod (WD-B, Wujin Weighing Co. Ltd). Parental height was measured with a stadiometer (Leicester height measure, Invicta Plastic Co. Ltd) with the same precision as the children’s. BMI (kg_/_m^2^) was calculated for parents and children. The BMI standard deviation score (BMI SDS) for children was generated according to the WHO Child Growth Standards 2006/7, which is an international applicable standard for child growth regardless their ethnicity, socio-economic status and type of feeding [23]. A second visit to the families was conducted after seven days to answer possible questions and to collect the questionnaires and accelerometers.

### Physical activity

The Actigraph GT3X+ is a watch-size (5 x 5 x 2 cm), light, and tri-axial accelerometer that measures acceleration in three orthogonal axes. A sampling rate of 30 Hertz was used. Initiating, downloading and analyzing were performed in the ActiLife software (version 6.2.5). To extract only daytime activity, sleeping records were used to estimate sleep time. To exclude nighttime sleep, the hours between 9:00 pm and 6:59 am were removed for children and hours between 10:00 pm and 05:59 am for parents. Time for daytime naps was not removed. Vector magnitude (VM) total counts were used to exclude invalid data. After visually inspecting the data [24], days with <1000 counts/minute and <600 counts/minute were removed from children and parents’ records, respectively, since it is unlikely that the monitor was worn those days. Children and parents with at least one valid day were included. Since no intensity cut-points are available for wrist worn Actigraphs in adults or one-year-olds, the outcome measures were averaged PA for weekdays, weekends, and by hour, expressed as average counts per minute (CPM) based on the VM for both children and parents. The VM has been found to give a better estimate of energy expenditure than output from the vertical axis [25].

### Variables

The main outcome variable was children’s average PA level. As main exposure variables, child gender (boy/girl) and BMI SDS, parental BMI and PA were used. Child and parental weight, length/height, and age, maternal weight gain, living space, type of child care (parents/others), ability to walk (yes/no), parental educational level (middle school or below/high school/college/university), parental BMI category (normal weight/overweight/obese), and household income (≤3000/3001-5000/5001-10000/≥10001) were used as confounding variables.

### Statistical analysis

Descriptive data are presented as mean and standard deviation (SD), median (range) or n (%) accordingly. The independent sample t-test and the chi-square test were used to assess differences between included and excluded families. Differences in average PA between risk groups (HR and LR) and between genders (boys and girls) were assessed using t-test. A Generalized Estimating Equations (GEE) model was used to assess the children and parental PA level by hour, and to further compare the hourly PA patterns among the three.

Univariate regression analysis was used to assess the crude associations between children’s PA level and the main exposures. A forward selection procedure was then conducted to select confounding variables. Maternal PA, maternal BMI, BMI SDS, maternal education level, paternal PA, and paternal BMI were included in the forward selection model sequentially with p<0.25 as the including criteria [26]. Since the ability to walk and type of child care may influence children’s PA levels, these were also included as confounders in the final model. The final multiple regression model included the variables child gender and BMI SDS, parental BMI and PA, maternal educational level, ability to walk and type of child care.

A *p*-value <0.05 was considered significant. The data analyses were performed using SAS software, Version 9.3 of the SAS System for Windows.

## Results

After excluding families with incomplete data on parental weight (n = 10), 289 families were allocated to either the HR (n = 123) or LR (N = 166) group. A total of 135 families were excluded because of either not receiving accelerometers (n = 41) or not using them (n = 94). Another 31 families were excluded because either the child’s or parents’ accelerometry data were absent. Finally, 123 families (66 HR and 57 LR) remained for the analysis. Children and fathers had an average of five days of valid PA data for analysis and mothers six days. The range of days with valid data was 1–7 for both children and parents. The included and excluded families did not differ in parental BMI, age and educational level, gestational weight gain, household income, children’s BMI SDS, ability to walk or age.

Participants’ characteristics are summarized in Table 1. No differences were found between the HR and LR groups for any baseline characteristics (*p* = 0.06–0.97), apart from the significantly higher parental weight and BMI in the HR group (*p*<0.001), which was in accordance with the study design. No children were attending preschool; all were taken cared for by the mother and/or father or grandparents (data not shown).

**Table 1:** Characteristics of children (n = 123), mothers (n = 121) and fathers (n = 122).

	High-risk		Low-risk		*p*
	Mean (SD^a^)	n (%)	Mean (SD)	n (%)	
**Children**		66 (54)		57 (46)	
Age (months)	12.3 (0.3)		12.4 (0.2)		0.06
Birth weight (kg)	3.38 (0.44)		3.28 (0.41)		0.23
Birth length (cm)	50.4 (1.7)		50.7 (2.1)		0.45
Weight at age one (kg)	10.6 (1.2)		10.2 (1.0)		0.08
Length at age one (cm)	77.5 (2.8)		77.1 (2.8)		0.49
BMI at age one (kg/m^2^)	17.6 (1.6)		17.2 (1.4)		0.12
BMI SDS^b^ at age one	0.58 (0.99)		0.34 (1.02)		0.19
Ability to walk (yes)		26 (40)		22 (39)	0.48
**Mothers**		64 (53)		57 (47)	
Age (years)	30.1 (3.4)		29.9 (4.3)		0.71
Weight (kg)	65.7 (10.5)		55.7 (8.7)		<.001
Height (cm)	160.8 (4.9)		161.6 (3.8)		0.35
BMI (kg/m^2^)	25.4 (3.6)		21.3 (2.9)		<.001
BMI category					<.001
Normal weight		25 (39)		47 (82)	
Overweight		22 (34)		10 (18)	
Obese		17 (27)		0 (0)	
Pregnancy weight gain (kg)	15.0 (5.4)		15.3 (5.5)		0.75
Pre pregnancy weight (kg)	59.6 (9.6)		53.2 (7.7)		<.001
Educational level					0.96
Middle school		7 (11)		5 (9)	
High school		8 (13)		6 (11)	
Collage/university		49 (77)		46 (81)	
Physical activity (CPM^c^)	1905 (587)		1936 (542)		0.77
**Fathers**		64 (53)		58 (48)	
Age (year)	31.2 (4.3		31.0 (4.7)		0.82
Weight (kg)	82.9 (9.1)		69.6 (11.3)		<.001
Height (cm)	173.9 (4.3)		173.9 (4.1)		0.97
BMI (kg/m^2^)	27.4 (2.7)		23.0 (3.6)		<.001
BMI category					<.001
Normal weight		9 (14)		38 (67)	
Overweight		24 (38)		19 (33)	
Obese		31 (48)		0 (0)	
Educational level					0.78
Middle school		8 (13)		4 (7)	
High school		14 (22)		12 (21)	
Collage/university		42 (66)		42 (72)	
Physical activity (CPM)	1556 (517)		1581 (453)		0.79
**Household income** (RMB/month)					0.21
Low (≦3 000)		3 (7)		5 (14)	
Middle (3 001–5 000)		7 (16)		11 (31)	
Upper middle (5 001–10 000)		25 (57)		16 (44)	
High (≥10 001)		9 (20)		4 (11)	
**Living space** (m^2^), median (range)	98 (38–160)		91 (30–200)		0.75

Note:

^a^ refers to standard deviation;

^b^ refers to body mass index standard deviation score;

^c^ refers to count per minute on the vector magnitude

The level of PA among the total sample of children and stratified by risk group and gender is described in Table 2. The averaged PA was 2157 CPM (SD = 519) and 1987 CPM (SD = 488) for children in the HR and LR groups (p = 0.06) respectively. There were no differences in the averaged PA between the risk groups (p = 0.06) or between genders (*p* = 0.23).

**Table 2:** PA levels (CPM^c^) of children, by risk group and gender (n = 123).

	Total sample		High-risk		Low-risk		*p*
	N	Mean (SD^a^)	N	Mean (SD)	N	Mean (SD)	
**Total sample**	123	2078 (510)	66	2157 (519)	57	1987 (488)	0.06
**Gender**							0.23
Boys	74	2124 (507)	40	2194 (508)	34	2041 (500)	0.20
Girls	49	2009 (512)	26	2100 (540)	23	1907 (469)	0.19

Note:

^a^ refers to standard deviation;

^c^ refers to count per minute on the vector magnitude.

**Fig 1:**
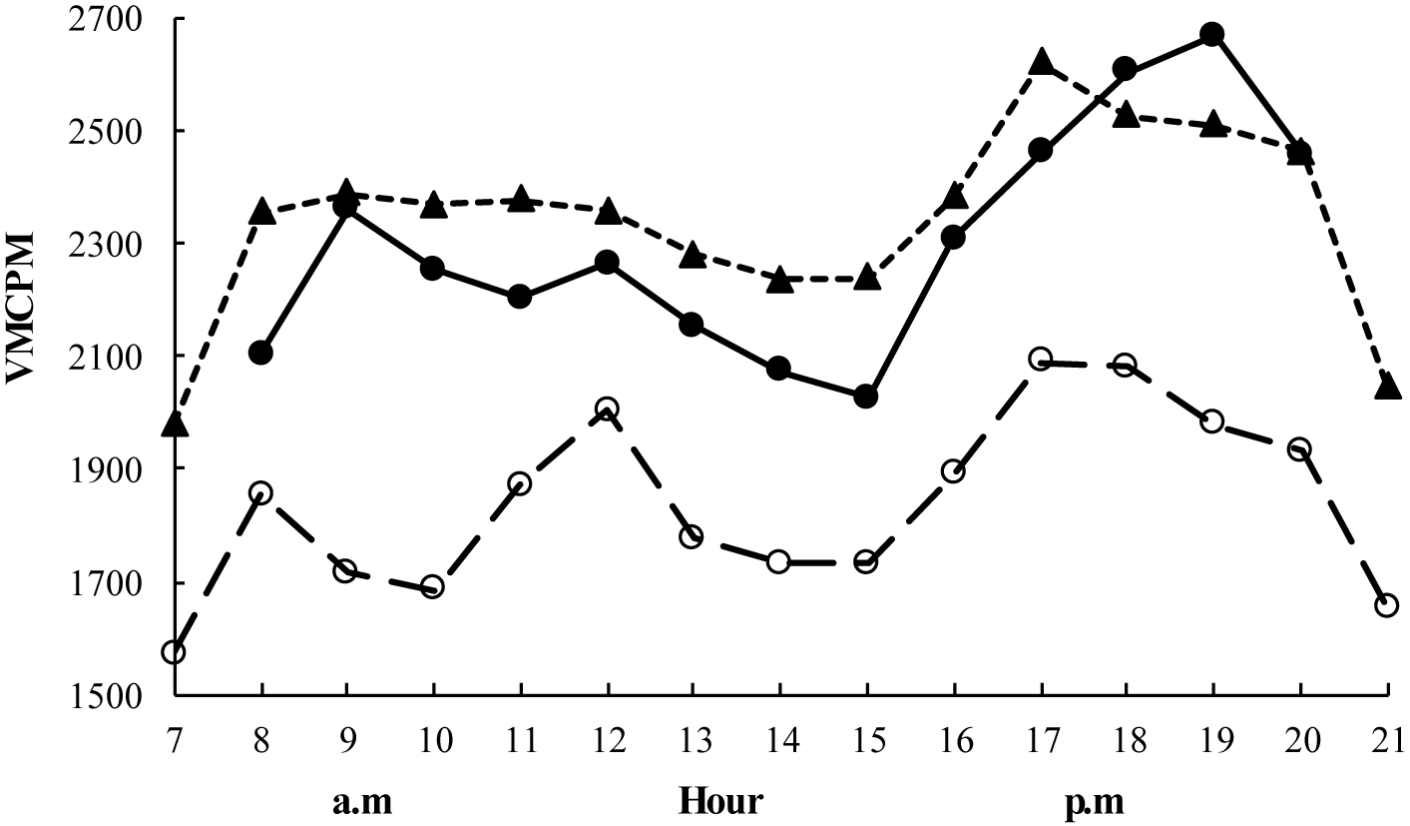
The hourly patterns of child and parental PA levels averaged over the measured days. Solid circles with solid line refers to children’s PA level, solid triangles with dashed line refers to mothers’ PA level, and blank circles with dashed line refers to fathers’ PA level. * Indicates significant differences between the lowest and highest mean activity level for children (*p*<0.001).

Fig 1 describes the hourly patterns of the averaged PA for children, mothers and fathers. Children’s average activity level was highest (2667 CPM) at 7 pm and lowest (2026 CPM) at 3 pm (*p*<0.001). Mothers and fathers had a similar pattern to their children, although the paternal activity level was consistently lower compared with the mothers’ (*p*<0.001) during the day. No difference in averaged PA was found between weekdays and weekends either for parents or for children. However, the hourly patterns revealed that children had a higher activity level at 6–8 pm (p<0.05) and fathers between 7–10 am (*p*<0.05) on weekends compared to weekdays. Maternal PA levels were similar during weekdays and weekends.

In the univariate analysis, neither child gender, BMI SDS, parental BMI or PA were associated with children’s PA level (*p*<0.05) (data not shown). In the multiple regression model (*R*^*2*^ = 0.19), only maternal PA was found to be associated with children’s PA level (*p* = 0.02), controlling for maternal educational level, ability to walk and type of child care (Table 3).

**Table 3:** The effect of gender, BMI SDS, parental BMI and PA on children’s PA.

	*Beta*	*Standard Error*	*p*
Child gender^d^	20.06	111.31	0.86
Child BMI SDS^b^	45.10	56.81	0.43
Maternal BMI (kg/m^2^)	16.19	15.40	0.30
Paternal BMI (kg/m^2^)	-8.69	15.41	0.57
Maternal PA (CPM^c^)	0.30	0.13	0.02
Paternal PA (CPM)	0.16	0.12	0.18
Maternal education level^e^	41.72	91.83	0.65
Ability to walk^f^	-94.60	115.76	0.42
Type of child care^g^	-160.73	174.05	0.36

Note:

^b^ refers to body mass index standard deviation score;

^c^ refers to count per minute on the vector magnitude;

^d^ boys was reference;

^e^ middle school or below was reference;

^f^ able to walk was reference;

^g^ parents look after child was reference.

## Discussion

To the best of our knowledge, this is the first study examining PA in one-year-old Chinese children with and without increased risk for obesity based on their parental weight status, using objective methods. Parental PA levels were also assessed and found to be similar during weekdays and weekends. Children were most active at 7 pm and fathers appeared to have a lower PA during all hours of the day compared to mothers and children. Child PA was not associated with any included variable, except for a positive associated with maternal PA.

PA level is related to age, primarily due to physical and motor development. Not all children are able to walk effectively at age one, which affects the degree of movement and the level of physically activity, detected with accelerometry [27]. In the present study about 40% of children had started to walk at age one. In addition, older children are usually taller and have longer limbs, which results in higher accelerations and thereby higher count values. This appears to be the most likely explanation for the lower PA counts observed in the children in the present study, compared with a previous study utilizing a wrist-worn accelerometer in two-year-old Swedish children who accumulated 3050 VM CPM on average [10]. As daytime sleep was not excluded in the current study, the time considered as physically active could be longer than the actual active time given the children’s age. So it would lower the outcome when presented as average counts per minute.

It is well known that boys are more physically active than girls, at least from four years of age onward [28, 29]. However, it remains unclear at what age these differences begin to occur, owing to the absence of related studies in younger children. According to the results from the present study, gender differences in PA are not apparent as early as in infancy. These results are in line with data from two-year-old Swedish children, where no differences in PA between boys and girls were found [10].

Children at high risk for obesity appeared to have higher PA levels than their counterparts at low risk, although the difference was insignificant. Living in larger home areas might contribute to the higher PA level of children in the HR group; in addition, we also suspected that parents with higher BMI might have been aware of the importance of PA and tended to encourage their children to be more physically active [30].

We found a significant maternal-child association in PA, which is consistent with earlier studies [12, 31, 32]. Although 92% children were cared for by grandparents during the daytime, most mothers undertook the child caring after work and at weekends. This may partly explain the association between maternal PA and child PA. Also, in the Melborne InFANT Program it was found that maternal PA when the child was 4 months old was positively associated with the child’s PA levels at 9- and 19-months[33]. This indicates that the mothers PA soon after delivery can impact the child PA in later childhood, also when the child is taken care of by others during daytime. Overall, our results denotes the importance of maternal PA for child PA during the early years of life.

Children had a marked increase in activity between 5 pm and 7 pm, peaking at 7 pm. Most Chinese parents finish work at 5 pm and supper is often served around 6 pm. The peak in PA at 7 pm can be explained by play time occurring after dinner. This pattern with a decrease in PA during mid–day and an increase during the late afternoon and early evening is consistent with studies on Australian 19-month-old children [34] and three-year-olds in the United States [35].

Although earlier studies on Chinese adults have shown only very small gender differences in PA examined on a non-family basis and using self-reported data [36, 37], we found Chinese fathers’ activity level to be consistently lower than that of mothers. A previous study has shown that men tend to be more sedentary than women at and after work [38]. Also, in order to get rid of the extra weight gained during pregnancy, women have been found to engage more in PA through daily activity at/after work [39]. In addition, Chinese women traditionally engage more in household duties than men, which could explain the PA peak between 5 and 6 pm among the mothers.

Accelerometers have mainly been worn and calibrated on the hip. However, there is a tendency that an increasing number of studies use wrist-worn accelerometers as it is more convenient and comfortable and improve compliance considerably [40–42]. In a study of 129 children aged 9-to-10 years, Fairclough et al showed that children preferred a wrist-worn accelerometer over a waist-worn one [17]. Studies assessing PA with wrist-worn accelerometers in children younger than two years is rare and it is important to highlight the fact that the outcome variable, expressed as CPM, is not comparable to results from studies where the monitor has been worn on other body sites.

This study is among the first to assess the PA level in one-year-old children, while most previous studies have focused on older children [43, 44]. The major strength is that PA was measured objectively over several days in both children and their parents and that anthropometrics were measured by trained research assistants.

Some limitations need to be addressed. First, day-time naps were not considered, which could result in an underestimation of PA based on the VM CPM, as mentioned earlier. The drop-out rate was large but no differences between completing and drop-out families were found. The PA level and pattern of the grandparents were not assessed. It is possible that their PA would have been significantly associated with the PA level of their grandchildren.

Regarding external validity, it should be highlighted that the families were selected from one of the largest cities in China. Compared to those working in the rural areas, sedentary occupations are more common in the cities, potentially affecting the average activity [45]. The sample size was relatively small. Also, the participating parents were well-educated, almost half of the mothers and more than 60% of the fathers finished collage/university. Further studies are needed to confirm the results in larger sample sizes and in diverse populations.

## Conclusions

This study shows that objectively measured PA in Chinese one-year-old children varies over the day, and the pattern is similar during weekdays and weekends. PA at this age is not correlated to gender, BMI SDS or parental weight status. The results need to be confirmed in larger scale and prospective studies with more contextual information to increase understanding of our findings.
